# Supplementary material for: Identification of Viruses in Molossus Bats from the Brazilian Amazon: A Descriptive Metagenomic Analysis
Source: Microorganisms. 2024 Mar 16;12(3):593. doi: 10.3390/microorganisms12030593 (PMC10974934; doi:10.3390/microorganisms12030593)
Supplement: Supplementary file 1 [file microorganisms-12-00593-s001.zip › microorganisms-2720596-supplementary.pdf]

Supplementary Material

Table S1 - Classification of the remaining readings - post treatment step - through the Kraken program.

Pools	Raw Reads	Classified	Chordate	Microbial	Bacteria	Virus	Fungi	Protozoa	Artificial	Not Classified
Pool 1	8,788,360	2,615,962	1,144,380	1,456,181	617,651	6,647	161,960	33,490	47	6,172,398
Pool 2	5,791,928	1,775,079	723,778	1,039,607	455,682	4,556	108,131	25,489	20	4,016,849
Pool 3	10,501,770	2,860,820	1,166,721	1,671,871	805,581	6,704	163,840	37,176	55	7,640,950
Pool 4	7,949,828	2,093,072	787,066	1,292,840	664,204	4,792	115,057	25,905	44	5,856,756
Pool 5	7,033,326	2,016,559	789,500	1,214,978	654,420	4,346	103,960	24,062	53	5,016,767
Pool 6	3,664,802	998,618	390,012	602,904	281,042	2,537	59,958	13,648	25	2,666,184
Pool 7	7,022,764	2,272,494	1,068,943	1,188,713	517,672	5,578	136,920	25,752	33	4,750,270
Pool 8	11,943,996	3,330,159	1,233,522	2,066,947	1,110,316	7,186	178,610	39,398	58	8,613,837
Pool 9	5,697,544	1,738,026	660,552	1,066,281	458,355	4,970	115,488	25,941	47	3,959,518
Pool 10	9,923,418	2,928,160	1,309,218	1,601,480	702,103	6,987	172,945	36,060	44	6,995,258
Pool 11	10,668,342	3,564,162	1,627,686	1,916,910	807,758	8,493	209,718	45,357	68	7,104,180
Pool 12	5,800,918	1,822,946	790,588	1,020,662	444,757	4,626	111,520	24,094	36	3,977,972
Pool 13	10,399,992	2,831,252	1,087,258	1,721,676	795,689	7,383	173,872	39,106	44	7,568,740
Total	105,186,988	30,847,309	12,779,224	1,7861,050	8,315,230	74,805	1,811,979	395,478	574	74,339,679

Table S2 - Nucleotide and amino acid identity matrix of the MBMV sequence with sequences from other Morbilliviruses.

	1	2	3	4	5	6	7	8	9	10	11
1 - *Molossus bat mobilivirus*		85.8	84.0	85.2	83.8	79.8	81.6	77.8	76.8	76.8	64.1
2 - *Porcine morbillivirus*	72.6		83.7	90.8	84.2	79.6	83.0	78.4	77.6	77.4	65.5
3 - *Canine morbillivirus*	72.1	61.3		83.4	93.0	81.8	83.8	80.2	79.0	79.0	64.1
4 - *Phyllostomus bat morbillivirus*	71.7	70.2	60.3		84.8	80.8	83.8	77.8	78.0	78.4	66.3
5 - *Phocine morbillivirus*	71.4	60.9	74.7	60.6		82.4	83.8	80.4	80.0	80.4	64.7
6 - *Cetacean morbillivirus*	69.4	58.8	61.1	59.2	61.1		79.0	80.8	77.0	81.2	63.5
7 - *Myotis bat morbillivirus*	69.3	57.3	59	57.5	58.7	57.8		79.2	79.2	80.2	64.5
8 - *Small ruminant morbillivirus*	68.9	57.1	58.3	57.1	58.2	61.6	56.6		82.4	83.0	65.3
9 - *Rinderpest morbillivirus*	68.7	57.0	58.0	57.2	58.2	60.5	56.7	62.2		88.6	62.5
10 - *Measles morbillivirus*	67.0	57.2	58.3	57.0	58.1	61.0	56.1	61.4	68.3		64.5
11 - *Feline morbillivirus*	60.6	46.7	46.9	47.0	47.8	47.7	47.2	46.0	46.5	46.4	

Legend: Blue – amino acid indentity values; Yellow – nucleotide identity values

Figure S1 - Eleven Morbilliviruses amino acid sequences alignment, including the *Molossus bat morbillivirus* sequence.

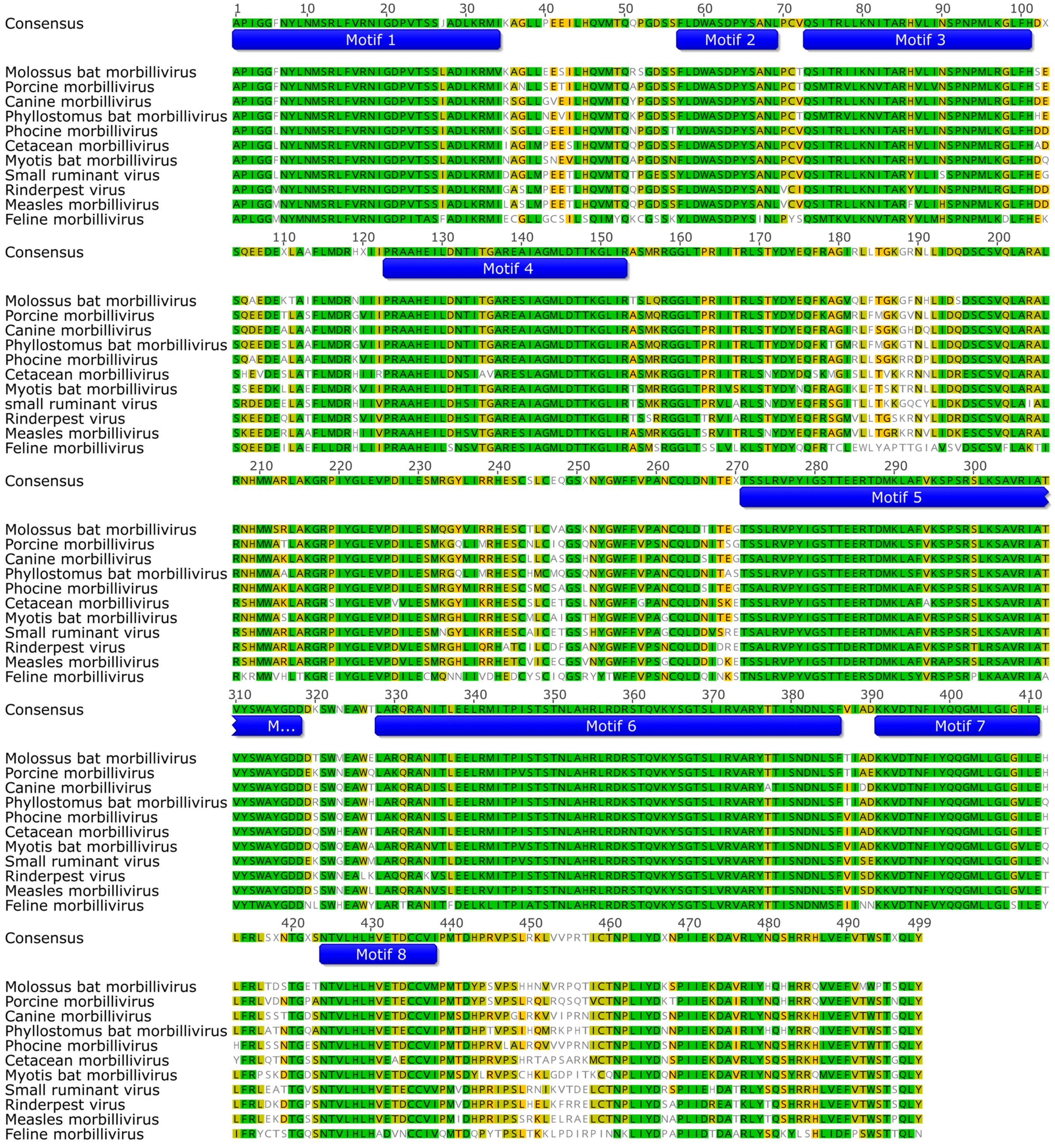

Legend: Annotations in blue highlight regions that appear to be the protein motifs of *Morbillivirus* genus. The motifs are regions with conserved amino acids, seen in green. Yellow indicates amino acid sites that are conserved in most, but not all, sequences. The regions in gray are those with highly divergent sites among the sequences

Table S3 - Nucleotide and amino acid identity matrix of *Culex dicistrovirus 2* 5RO20/CDV with sequences of other Cripaviruses.

	1	2	3	4	5	6
1 - *Culex dicistrovirus 2* 5R020/CDV		94.3	918	42.2	24.7	23.8
2 - *Culex dicistrovirus* 2	89.5		96.6	43.1	25.0	23.8
3 - *Rhopalosiphum padi virus*	83.4	90.9		42.9	25.6	24.4
4 - *Aphid lethal paralysis virus*	51.7	52.3	52.6		24.3	25.6
5 - *Cricket paralysis virus*	35.8	36.4	36.4	34.2		56.3
6 - *Drosophila C virus*	35.2	36.3	36.4	35.5	59.3	

Legend: Blue - amino acid identity values; Yellow - nucleotide identity values.

Figure S2 - Alignment of the amino acid sequences of six Dicistroviruses, including the *Culex dicistrovirus 2* 5RO20/CDV sequence.

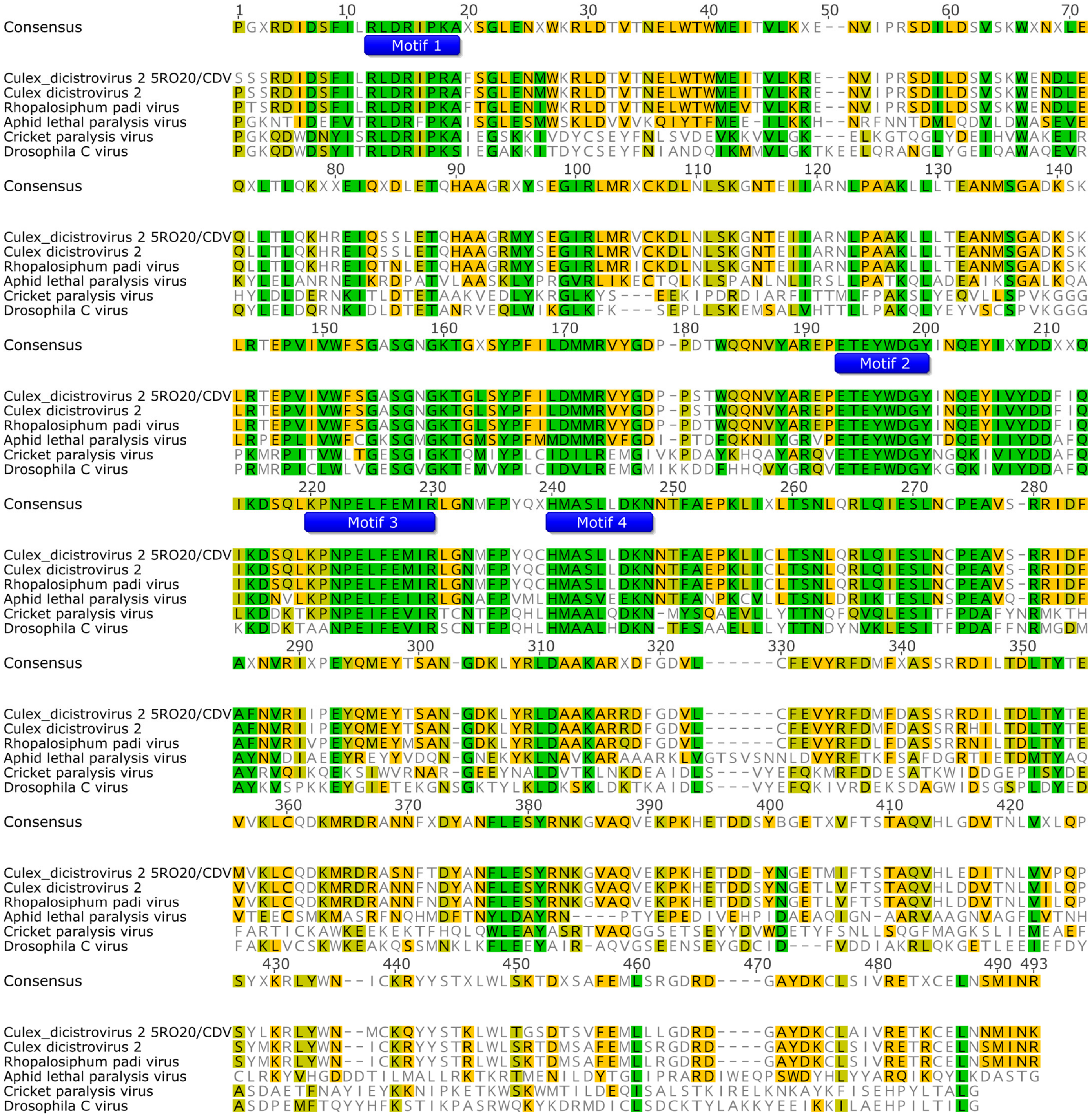

Legend: Blue annotations highlight regions that appear to be the protein motifs of the *Cripavirus* genus. The motifs are regions with conserved amino acids, seen in green. In yellow we see amino acid sites that are conserved in most, but not all, sequences. The regions in gray are those with very divergent sites between the sequences.
